# Depletion of platelets enhances therapeutic antitumor effects generated by therapeutic DNA vaccine

**DOI:** 10.1186/2051-1426-1-S1-P222

**Published:** 2013-11-07

**Authors:** Sung Yong Lee, Tae Heung Kang, Jayne Knoff, Chien-Fu Hung, Tc Wu, Kyung Ho Kang, Jae Jeong Shim

**Affiliations:** 1 Internal Medicine, Korea University Medical Center, Seoul, Republic of Korea; 2 Immunology, Konkuk University Medical Center, Chungju, Republic of Korea; 3 Patholgy, Johns Hopkins Medical Institutions, Baltimore, MD, USA; 4 Obstetrics and Gynecology, Johns Hopkins Medical Institutions, Baltimore, MD, USA; 5 Oncology, Johns Hopkins Medical Institutions, Baltimore, MD, USA

## 

It has been well established that platelets are associated with tumor progression and metastasis as they can support angiogenesis and prevent hemorrhage. As such, platelets may be an ideal target for enhancing anticancer immunotherapies. Thus, we hypothesized that inducing thrombocytopenia would increase the leakiness of tumor vasculature thereby facilitating the effective delivery of antigen-specific T cells generated by DNA vaccination to tumor loci in order to induce CD8+ T cell-mediated tumor killing. We have previously developed a therapeutic HPV DNA vaccine encoding calreticulin (CRT) linked to HPV-16 E7 antigen (CRT/E7) to enhance therapeutic DNA vaccine potency. In the current study, we explore the employment of a platelet-depleting antibody or heparin in combination with CRT/E7 DNA vaccine in a mouse model for its potential to enhance E7-specific CD8+ T cell immune responses as well as antitumor effects against E7-expressing tumors. We induced severe acute thrombocytopenia in mice bearing subcutaneous TC-1 tumors. TC-1 tumor cells originate from the mouse lung and express the HPV-16 E7 tumor-specific antigen. In a TC-1 mouse tumor model, platelet depletion increased E7-specific CD8+ T cells in the peripheral blood and reduced the growth of the tumor mass following CRT/E7 DNA vaccination. Next, we isolated platelet-rich plasma from tumor-bearing and naïve mice. When TC-1 tumor cells were coincubated with E7-specific CD8+ T cells under platelet-rich plasma and found that E7-specific CD8+ T cells had decreased IFN-γ secretion in the presence of platelet-rich plasma from tumor-bearing mice compared to that of naïve mice. Furthermore, when incubated with E7-specific CD8+ T cells and platelets, TC-1 cells were killed significantly less efficiently compared to those incubated with E7-specific CD8+ T cells alone. Importantly, we found that GFP-tagged TC-1 cells incubated with platelets were coated by the platelets, which were subsequently activated (CD26P+). Taken together, these data suggest that platelet depletion increases tumor-specific CD8+ T cells and enhances their tumor killing effects. Furthermore, This study provides proof in principle that platelets directly inhibit CD8+ T cells from killing tumor cells. In conclusion, platelet depletion or heparin therapy can stimulate antitumor immune responses and this treatment methodology may be applicable to a variety of cancers.

